# Nationwide Molecular Epidemiology of Measles Virus in Japan Between 2008 and 2017

**DOI:** 10.3389/fmicb.2019.01470

**Published:** 2019-07-04

**Authors:** Fumio Seki, Masahiro Miyoshi, Tatsuya Ikeda, Haruna Nishijima, Miwako Saikusa, Masae Itamochi, Hiroko Minagawa, Takako Kurata, Rei Ootomo, Jumboku Kajiwara, Takashi Kato, Katsuhiro Komase, Keiko Tanaka-Taya, Tomimasa Sunagawa, Kazunori Oishi, Nobuhiko Okabe, Hirokazu Kimura, Shigeru Suga, Kunihisa Kozawa, Noriyuki Otsuki, Yoshio Mori, Komei Shirabe, Makoto Takeda, Measles Virus Surveillance Group of Japan, Rika Komagome, Technical Support Team for Measles Control in Japan, Kenji Someya

**Affiliations:** Niigata City Inst. of P.H.E.; Ibaraki Pref. Inst. of P.H.; Tochigi Pref. Inst. of P.H.E.S.; Gunma Pref. Inst. of P.H.E.S.; Saitama Inst. of P.H.; Saitama City Inst. of Health Sci. and Res.; Tokyo Metropolitan Inst. of P.H.; Chiba Pref. Inst. of P.H.; Chiba City Inst. of Health and Environ.; Kanagawa Pref. Inst. of P.H.; Kawasaki City Inst. of P.H.; Sagamihara City Inst. of P.H.; Yamanashi Inst. for P.H.; Conservation Res. Inst.; Shizuoka Inst. of Environ. and Hygiene; Shizuoka City Inst. of Environ. Sci. and P.H.; Ishikawa Pref. Inst. of P.H.E.S.; Aichi Pref. Inst. of P.H.; Hamamatsu City Health Environ. R.C.; Nagoya City P.H. Res. Inst.; Tokushima Pref. P.H., Pharmaceutical and E.S. Center; Gifu Pref. Res. Inst. for Health and E.S.; Gifu City Inst. of P.H.; Nagano City Health Center; Mie Pref. Health and Environ. Res. Inst.; Shiga Pref. Inst. of P.H.; Kyoto Pref. Inst. of P.H.E.; Kyoto City Inst. of Health and E.S.; Osaka Inst. of P.H. (formerly Osaka Pref. Inst. of P.H.); Osaka Inst. of P.H. (formerly Osaka City Inst. of P.H.E.S); Sakai City Inst. of P.H.; Hyogo Pref. Inst. of P.H.E.S.; Kobe Inst. of Health; Himeji City Res. Inst. of P.H.; Amagasaki City Inst. of P.H.; Nara Pref. Inst. of Health; Wakayama City Inst. of P.H.; Shimane Pref. Inst. of P.H.E.S.; Okayama Pref. Inst. for E.S. and P.H.; Center for P.H.E., Hiroshima Pref. Technology Res. Inst.; Hiroshima City Inst. of P.H.; Yamaguchi Pref. Inst. of P.H.E.; Kagawa Pref. Res. Inst. for E.S. and P.H.; Fukuoka Inst. of Health and E.S.; Fukuoka City Inst. of Health and Environ.; Kitakyushu City Inst. of E.S.; Oita Pref. Inst. of Health and Environ.; Kumamoto Pref. Inst. of P.H. and E.S.; Kumamoto City Environ. P.H., Public Health; P.H.E., Public Health and Environment; P.H.E.S, Public Health and Environmental Sciences/Public Health and Environmental Science; E.S., Environmental Sciences/Environmental Science; R.C., Research Center; Inst., Institute; Pref., Prefectural; Res., Research. Department of Virology 3, NIID; Infectious Disease Surveillance Center, NIID; ^1^Department of Virology 3, National Institute of Infectious Diseases, Tokyo, Japan; ^2^Hokkaido Institute of Public Health, Sapporo, Japan; ^3^Yamagata Prefectural Institute of Public Health, Yamagata, Japan; ^4^Chiba Prefectural Institute of Public Health, Chiba, Japan; ^5^Yokohama City Institute of Public Health, Yokohama, Japan; ^6^Toyama Institute of Health, Imizu, Japan; ^7^Aichi Prefectural Institute of Public Health, Nagoya, Japan; ^8^Osaka Institute of Public Health, Osaka, Japan; ^9^Tottori Prefectural Institute of Public Health and Environmental Science, Tottori, Japan; ^10^Fukuoka Institute of Health and Environmental Sciences, Dazaifu, Japan; ^11^Okinawa Prefectural Institute of Health and Environment, Uruma, Japan; ^12^Infectious Disease Surveillance Center, National Institute of Infectious Diseases, Tokyo, Japan; ^13^Kawasaki City Institute for Public Health, Kawasaki, Japan; ^14^Graduate School of Health Science, Gunma Paz University, Takasaki, Japan; ^15^Department of Pediatrics, National Mie Hospital, Tsu, Japan; ^16^Graduate School of Medicine, Yokohama City University, Yokohama, Japan; ^17^Yamaguchi Prefectural Institute of Public Health and Environment, Yamaguchi, Japan

**Keywords:** measles virus, molecular epidemiology, genotype, elimination, Japan

## Abstract

Genotyping evidence that supports the interruption of endemic measles virus (MV) transmission is one of the essential criteria to be verified in achieving measles elimination. In Japan since 2014, MV genotype analyses have been performed for most of the measles cases in prefectural public health institutes nationwide. With this strong molecular epidemiological data, Japan was verified to have eliminated measles in March, 2015. However, even in the postelimination era, sporadic cases and small outbreaks of measles have been detected repeatedly in Japan. This study investigated the nationwide molecular epidemiology of MV between 2008 and 2017. The 891 strains in the total period between 2008 and 2017 belonged to seven genotypes (D5, D4, D9, H1, G3, B3, and D8) and 124 different MV sequence variants, based on the 450-nucleotide sequence region of the N gene (N450). The 311 MV strains in the postelimination era between 2015 and 2017 were classified into 1, 7, 8, and 32 different N450 sequence variants in D9, H1, B3, and D8 genotypes, respectively. Analysis of the detection period of the individual N450 sequence variants showed that the majority of MV strains were detected only for a short period. However, MV strains, MVs/Osaka.JPN/29.15/ [D8] and MVi/Hulu Langat.MYS/26.11/ [D8], which are named strains designated by World Health Organization (WHO), have been detected in many cases over 2 or 3 years between 2015 and 2017. The WHO-named strains have circulated worldwide, causing outbreaks in many countries. Epidemiological investigation revealed repeated importation of these WHO-named strains into Japan. To demonstrate the elimination status (interruption of endemic transmission) in situations with repeated importation of the same strains is challenging. Nevertheless, the detailed sequence analysis of individual MV strains and chronological analysis of these strains provided sufficient evidence to show that Japan has still maintained its measles elimination status in 2017.

## Introduction

In 2005, the WHO member states in the Western Pacific Region (WPR) decided to target the elimination of measles by 2012 in this region. Aiming to eliminate measles in Japan, the Ministry of Health, Labour and Welfare, Japan (MHLW) announced the Special Infectious Disease Prevention Guidelines on Measles (MHLW Measles Guideline) and requested mandatory reporting of all measles cases (i.e., case-based surveillance) since January 2008. In November 2010, to strengthen the surveillance, MHLW issued a notice by the Director of Tuberculosis and Infectious Diseases Control Division in MHLW (Director’s Notice), requesting local governments to conduct measles virus (MV) testing by PCR for all suspected measles cases as possible. The MHLW Measles Guideline was revised in April 2013, further requesting local governments to conduct MV detection testing by PCR. The detection of MV was critically important to obtain genotyping evidence that supports the interruption of endemic MV transmission, one of the essential criteria to be verified in achieving measles elimination (World Health Organization [WHO] and Regional Office for the Western Pacific [WPRO], 2013). The variable 450-nucleotide sequence region of the MV nucleocapsid gene (N450) has been generally used for genotyping and monitoring individual MV strains (World Health Organization [WHO], 2015). These MV detection and genotyping data were included in the annual report by the National Verification Committee (NVC) for measles elimination in Japan. After the careful review of the NVC annual report, the Regional Verification Commission (RVC) for measles elimination in WPR verified that Japan achieved measles elimination on March 27, 2015. This study focuses on the molecular epidemiology of MV between 2008 and 2017 in Japan.

## Materials and Methods

### Nucleotide Sequencing of the 450-Nucleotide Region in the N Gene

According to the MHLW Measles Guideline under the Infectious Diseases Control Law in Japan, clinical samples (including throat swab, blood, and urine samples) were collected from measles-suspected cases and sent to PHIs to detect MV. The criteria for measles-suspected cases were appearance of two or three clinical signs of fever, rash, and either cough, coryza, or conjunctivitis. Samples, which were positive for MV RNA by reverse-transcription PCR (RT-PCR), were subjected to nucleotide sequencing of the WHO-recommended 450-nucleotide region in the N gene (N450) (nucleotide positions 1233–1682). The region encodes the carboxyl-terminal 150 amino acids of the nucleoprotein. Briefly, the extracted viral RNAs were reverse-transcribed into cDNAs using a commercially available reverse transcription kit and random hexamer primers. The nucleotide region containing the N450 region was amplified by nested PCR. The first PCR primer set was pMvGTf1m (5′-CGRTCTTACTTYGATCCRGC-3′) and pMvGTr1 (5′-TTATAACAATGATGGAGG-3′), and the nested PCR primer set was pMvGTf2m (5′-AGAYTAGGRCARGAGATGGT-3′) and pMvGTr2 (5′-GAGGGTAGGCGGATGTTGTT-3′). After purification of the fragment, the nucleotide sequence was determined by a fluorescent dye-terminator cycle sequencing method using the primers, pMvGTf2m or pMvGTr2.

### Phylogenetic Analysis

Phylogenetic analysis was conducted using the MEGA program (version 7.0.6). In addition to the MV strains detected in Japan, WHO reference MV strains, the WHO-named strains, and MV strains detected outside of Japan (overseas MV strains) and having the identical sequences to the MV strains detected in Japan were included in this analysis (Supplementary Table 1). The list of MV strains used in this study and DDBJ/EMBL/GenBank accession numbers of these strains are shown in Supplementary Table 1. The overseas MV strains having the identical sequences to the MV strains detected in Japan were searched by using WHO Measles Nucleotide Surveillance Database (MeaNS^1^) (WHO and PHE). Phylogenetic trees were constructed by the maximum-likelihood method using the Tamura–Nei model. The reliability of the tree at each branch node was assessed by the bootstrap method with 1,000 replicates. The genotype of MV strains was determined based on the phylogenetic tree topology constructed with the WHO reference strains.

## Results and Discussion

### The Global Situation Towards Measles Elimination

In 2012, the Global Vaccine Action Plan (GVAP) was endorsed by the World Health Assembly (World Health Organization [WHO], 2013a). GVAP set a goal to eliminate measles and rubella in at least five WHO regions by 2020 by strengthening immunization programs (World Health Organization [WHO], 2013a). As a result of the global effort on immunization, the number of reported measles cases has declined greatly, resulting in a 80% decrease in measles deaths between 2000 and 2017. The region of the America has declared measles elimination in this region in 2016. Thirty-seven countries (70%) in European region have been also verified as having achieved measles elimination in 2017. In the South-East Asia Region, two countries (Bhutan and Maldives) have achieved measles elimination in 2017. On the other hand, no countries has achieved measles elimination in the Eastern Mediterranean and Africa regions. Japan is a member state in WPR. In 2014, RVC in WPR has verified that Australia, Macao (China), Mongolia, and the Republic of Korea have achieved measles elimination (World Health Organization [WHO] and Regional Office for the Western Pacific [WPRO], 2014). In addition to the four countries and areas, Japan, Brunei, and Cambodia were verified as having achieved measles elimination in 2015 (World Health Organization [WHO] and Regional Office for the Western Pacific [WPRO], 2015). However, since 2015 a large measles outbreak occurred in Mongolia, and endemic MV transmission has been re-established in Mongolia (World Health Organization [WHO] and Regional Office for the Western Pacific [WPRO], 2016). In 2017, Japan and seven countries or areas [Australia, Brunei, Cambodia, Hong Kong (China), Macao (China), New Zealand, and South Korea] have been verified as having achieved or sustained measles elimination (World Health Organization [WHO], Regional Office for the Western Pacific [WPRO], 2017; Dabbagh et al., 2018).

As just described, we have made great progress toward measles elimination by improving vaccination coverages. However, considerable variations in vaccination coverages exist among regions, countries, and areas, causing measles outbreaks continuously somewhere in the world. Even a small reduction of vaccination rate due to vaccine hesitancy may result in outbreaks of measles (Lo and Hotez, 2017).

### Performance of MV Detection and Genotype Analysis

As described above, Japan started case-based surveillance of measles from 2008 in accordance with the MHLW Measles Guideline. At that time, laboratory testing was not mandatory to confirm a clinical diagnosis of measles, but many cases were laboratory-confirmed by detecting MV-specific serum IgM. In Japan, serum specimens are sent to commercial laboratories for detection of measles-specific IgM by ELISA. By contrast, the percentage of measles cases confirmed by PCR was only 0.7–4.7% between 2008 and 2010 (Table 1). Clinical specimens must be sent to PHIs for virus detection by RT-PCR. After the Director’s Notice was issued in November 2010, the ratio of measles cases with virus detection data provided by PCR increased from 23.2 to 28.3% between 2011 and 2013. This ratio further increased from 71.4 to 97.8% between 2014 and 2017 after the revised MHLW Measles Guideline was put into force in April 2013 (Table 1). The ratio of the cases with virus detection data provided by PCR was 84.5% (387 out of 458 cases) in 2014. When MV is detected by RT-PCR, PHIs (or NIID) then conduct genotype analysis. The great majority of cases, which were positive for RT-PCR, had the genotyping data for MV, which have provided strongly supportive evidence for interruption of endemic MV transmission (measles elimination) in Japan by 2015. The total number of measles cases in 2015 was only 35 (Table 1), which was a record low in the history of Japan. However, several MV epidemics triggered by imported measles cases were recorded in 2016 and 2017, which resulted in 165 and 185 MV cases in Japan. To assess the measles elimination status in Japan, MV genotype data were obtained from most of the cases in 2016 and 2017 (140 and 181 cases out of 165 and 185 cases in 2016 and 2017, respectively) (Table 1).

**Table 1:** The number of reported measles cases and MV genotype data in Japan.

Year	The total number of reported measles cases^∗^	The number of lab-confirmed measles cases by PCR	% of PCR detection	The number of cases with MV genotype data							
				Total	Genotype						
					D5	D4	D9	H1	G3	B3	D8
2008	11,023	258	2.3	43	39	1	0	3	0	0	0
2009	724	5	0.7	2	0	0	1	0	0	0	1
2010	443	21	4.7	20	0	1	16	2	0	0	1
2011	438	124	28.3	115	0	58	49	0	2	0	6
2012	282	72	25.5	41	0	5	12	7	0	0	17
2013	233	54	23.2	50	0	0	5	5	0	28	12
2014	458	387	84.5	309	0	0	21	13	0	220	55
2015	35	25	71.4	25	0	0	4	5	0	4	12
2016	165	140	84.8	122	0	0	0	56	0	1	65
2017	185	181	97.8	164	0	0	0	2	0	7	155

*^∗^The total number of cases in each year is counted based on the epidemiological week.*

### Overview of MV Genotypes Detected in Japan

Based on sequence variations in the N450 region, the MV strains are currently classified into 24 genotypes (A, B1–B3, C1, C2, D1–D11, E, F, G1–G3, H1, and H2) (World Health Organization [WHO], 2015). However, only six genotypes (B3, D4, D8, D9, G3, and H1) are currently circulating globally, and many other genotypes have not been detected for >10 years. From 1990 D5 strains were endemically circulated in Japan (Takahashi et al., 2000; Okafuji et al., 2006; Morita et al., 2007; Taira et al., 2008; Nagai et al., 2009; Nagano et al., 2011; Kaida et al., 2018), but no D5 strain was detected in Japan in recent years (Table 1). Measles outbreaks by genotype D4 strains occurred between 2010 and 2012 in Japan (Table 1). In total, 64 genotype D4 strains (1, 58, and 5 strains in 2010, 2011, and 2012, respectively) were detected. Many D4 MV cases during these outbreaks in Japan had an epidemiological linkage (travel history) to European countries (National Institute of Infetious Diseases [NIID], and Tuberculosis, and Infectious Disease Control Division, Ministry of Health, Labor, and Welfare [MHLW], 2012a,b; Nadaoka et al., 2014; Kaida et al., 2018). The MV genotyping data also suggested that between 2010 and 2012, Japan has experienced many imported measles cases from European countries. Importation of the genotype D4 strain from New Zealand was also detected in 2011 (Watanabe et al., 2012). The transmission of genotype D4 strains was completely interrupted after 2013 in Japan (Table 1).

Many genotype D9 strains were detected in Japan between 2009 and 2015 (Table 1; Aoki et al., 2009). They were detected every year. Between 2010 and 2011, large D9 outbreaks have occurred in the Philippines (Centeno et al., 2015; Takashima et al., 2015), and many cases in Japan had travel history to the Philippines (National Institute of Infetious Diseases [NIID], and Tuberculosis and Infectious Disease Control Division, Ministry of Health, Labor and Welfare [MHLW], 2011a,b; National Institute of Infetious Diseases [NIID], and Tuberculosis, and Infectious Disease Control Division, Ministry of Health, Labor, and Welfare [MHLW], 2012a,b; Centeno et al., 2015). The sequence analysis of MV strains has confirmed that certain D9 strains in Japan have the identical N450 nucleotide sequences to the Philippine D9 strains (Centeno et al., 2015). The D9 strains were first detected in the Philippines in 2007 and have been predominant until 2012 in the Philippines (Fuji et al., 2011; Centeno et al., 2015; Takashima et al., 2015). Outbreaks between 2010 and 2011 in the Philippines were presumably caused by several D9 strains with different N450 sequences (Centeno et al., 2015). D9 virus was also exported from the Philippines to Taiwan and Australia in 2010 and 2011, respectively (Jayamaha et al., 2012; Cheng et al., 2013). In Japan, importation of D9 strains from other Asian countries, such as Malaysia (National Institute of Infetious Diseases [NIID], and Tuberculosis, and Infectious Disease Control Division, Ministry of Health, Labor, and Welfare [MHLW], 2012a,b; Watanabe et al., 2012), Indonesia (National Institute of Infetious Diseases [NIID], and Tuberculosis, and Infectious Disease Control Division, Ministry of Health, Labor, and Welfare [MHLW], 2012a,b), and Thailand (National Institute of Infetious Diseases [NIID], and Tuberculosis, and Infectious Disease Control Division, Ministry of Health, Labor, and Welfare [MHLW], 2012a,b), were also detected in 2011. In 2011, as observed in Japan, genotype D9 strains were also predominant strains in Taiwan, Singapore, and New South Wales (Rosewell et al., 2012; Cheng et al., 2013; Ho et al., 2014), where endemic transmission of MV has been eliminated or well controlled. In 2011, South Korea has also experienced imported D9 cases, although the origin was unclear (Park et al., 2013). These data suggest that between 2010 and 2011, genotype D9 strains have been spread from the Philippines or other endemic countries across Asian countries, including Japan. Indeed, many cases had travel histories abroad (National Institute of Infetious Diseases [NIID], and Tuberculosis and Infectious Disease Control Division, Ministry of Health, Labor and Welfare [MHLW], 2011a,b; National Institute of Infetious Diseases [NIID], and Tuberculosis, and Infectious Disease Control Division, Ministry of Health, Labor, and Welfare [MHLW], 2012a,b). The transmission of genotype D9 strains was completely interrupted in Japan (Table 1).

Genotype H1 strains were detected in Japan almost every year (Table 1). For >20 years, genotype H1 strains endemically circulated in China (Xu et al., 2014; Hagan et al., 2018). Imported genotype H1 MV cases were often detected in Japan (National Institute of Infetious Diseases [NIID], and Tuberculosis and Infectious Disease Control Division, Ministry of Health, Labor and Welfare [MHLW], 2013a,b; National Institute of Infetious Diseases [NIID], and Tuberculosis, and Infectious Disease Control Division, Ministry of Health, Labor, and Welfare [MHLW], 2015a,b, 2016a,b; National Institute of Infetious Diseases [NIID], and Tuberculosis and Infectious Disease Control Division, Ministry of Health, Labor and Welfare [MHLW], 2017a,b; Minagawa et al., 2015; Miyoshi et al., 2017). In total, 93 genotype H1 strains were detected between 2008 and 2017 (Table 1). Only two genotype G3 strains were detected in Japan in the period between 2008 and 2017. They were imported from Indonesia (Tanaka et al., 2011; National Institute of Infetious Diseases [NIID], and Tuberculosis, and Infectious Disease Control Division, Ministry of Health, Labor, and Welfare [MHLW], 2012a,b).

In Japan, many genotype B3 strains were detected between 2013 and 2014, while only small numbers of genotype B3 strains were detected after 2015 (Table 1). In total, 260 genotype B3 strains were detected between 2013 and 2017 (Table 1). In 2014, large measles outbreaks have occurred in the Philippines (Takashima et al., 2015) and many measles cases in 2014 in Japan had travel history to the Philippines (Takahashi et al., 2014; Minagawa et al., 2015; Miyoshi et al., 2015; National Institute of Infetious Diseases [NIID], and Tuberculosis, and Infectious Disease Control Division, Ministry of Health, Labor, and Welfare [MHLW], 2015a,b; Yagi et al., 2015; Miyoshi et al., 2017; Kaida et al., 2018). Imported cases from the Philippines were also reported in Korea (Park et al., 2017) and many European countries (Nic Lochlainn et al., 2016; Santibanez et al., 2017).

In recent years, genotype D8 has been one of the major genotypes detected in Japan (Table 1). In 2017, the great majority of MV strains detected in Japan were genotype D8 strains (Table 1). In total, 324 genotype D8 strains were detected between 2009 and 2017. In 2014, a large outbreak of the D8 genotype strain occurred in Vietnam (Pham et al., 2014) and seven imported D8 cases from Vietnam were detected in Japan that year (National Institute of Infetious Diseases [NIID], and Tuberculosis, and Infectious Disease Control Division, Ministry of Health, Labor, and Welfare [MHLW], 2015a,b). Many imported D8 cases from Indonesia were also reported in 2013 (Kaida et al., 2018), 2014 (Miyoshi et al., 2015; National Institute of Infetious Diseases [NIID], and Tuberculosis, and Infectious Disease Control Division, Ministry of Health, Labor, and Welfare [MHLW], 2015a,b), 2015 (National Institute of Infetious Diseases [NIID], and Tuberculosis, and Infectious Disease Control Division, Ministry of Health, Labor, and Welfare [MHLW], 2016b), 2016 (National Institute of Infetious Diseases [NIID], and Tuberculosis and Infectious Disease Control Division, Ministry of Health, Labor and Welfare [MHLW], 2017a), and 2017 (Komabayashi et al., 2018; National Institute of Infetious Diseases [NIID], and Tuberculosis and Infectious Disease Control Division, Ministry of Health, Labor and Welfare [MHLW], 2018a,b). Thailand was also a major source of genotype D8 MV importation into Japan in 2012 (National Institute of Infetious Diseases [NIID], and Tuberculosis and Infectious Disease Control Division, Ministry of Health, Labor and Welfare [MHLW], 2013a), 2016 (National Institute of Infetious Diseases [NIID], and Tuberculosis and Infectious Disease Control Division, Ministry of Health, Labor and Welfare [MHLW], 2017a,b), and 2017 (National Institute of Infetious Diseases [NIID], and Tuberculosis and Infectious Disease Control Division, Ministry of Health, Labor and Welfare [MHLW], 2018a,b).

### Detailed Analysis of Each Genotype

One or two reference strains have been set for each genotype. However, using only the genotype classification and reference strains is currently insufficient for monitoring the global transmission of MV. Considering this situation, WHO has set a certain number of “named strains” in addition to the reference strains. These named strains represent epidemiologically significant MV strains that circulate in multiple countries (World Health Organization [WHO], 2015).

### Genotype D5

**FIGURE 1:**
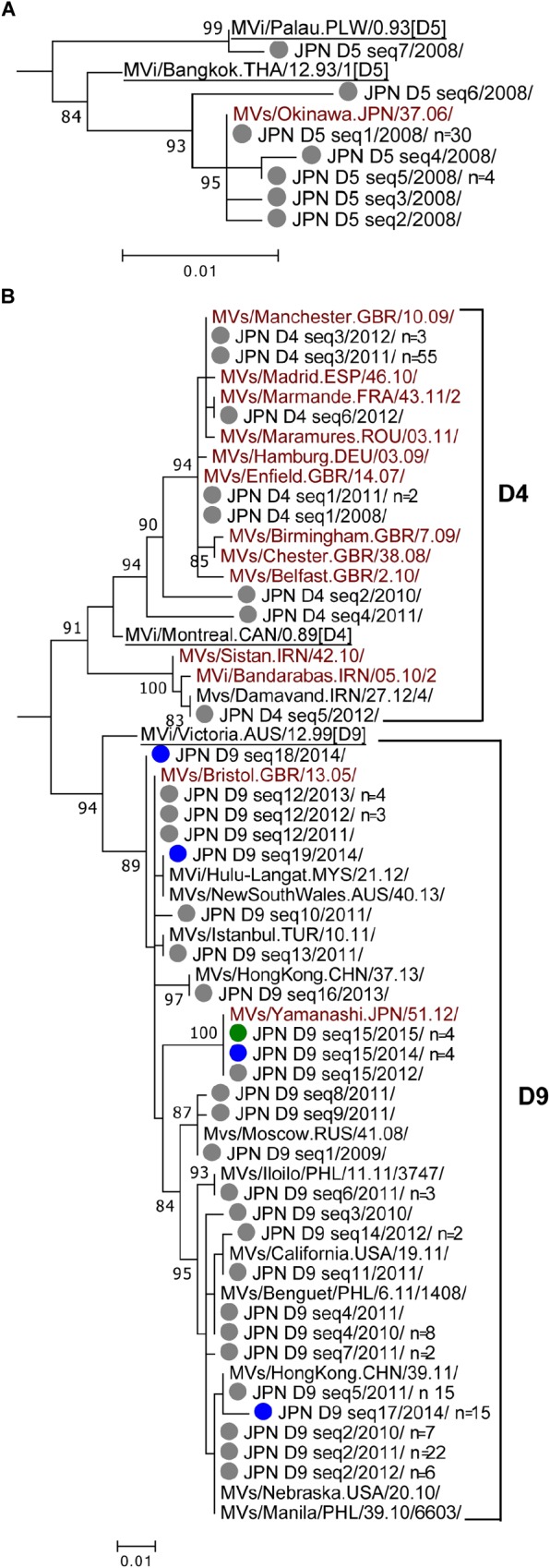
Phylogenetic trees of the genotype D5, D4, and D9 MV strains. The phylogenetic trees were constructed using the maximum-likelihood method based on the N450 nucleotide sequence. Circles colored in gray indicate strains detected in Japan between 2008 and 2013. Circles colored in blue, green, orange, and red indicate strains detected in Japan in 2014, 2015, 2016, and 2017, respectively. Underlined MV strains are WHO reference strains. MV strains with brown characters are WHO-named strains. Others are overseas MV strains with the identical N450 sequences to one or another MV strains detected in Japan. The number (*n*) of each MV strain with an identical N450 sequence is shown, if the strain was detected from more than one case. **(A)** Genotype D5. **(B)** Genotypes D4 and D9.

Based on the nucleotide sequence difference in the N450 region, the 39 D5 strains detected in 2008 in Japan (Table 1) were divided into seven different sequence variants (D5 seq1 to D5 seq7 variants [D5 seq1–7]). All D5 strains, except for the single D5 seq7 variant, were in the cluster of the MVi/Bangkok.THA/12.93/1[D5] reference strain. Among them, D5 seq1 variant (*n* = 30, 79%) had the identified N450 sequence to the MVs/Okinawa.JPN/37.06/ named strain (D5-Okinawa), which was detected in 2006, Okinawa, Japan. The phylogenetic tree data of MV strains in Japan suggested that the D5 seq2, D5 seq3, D5 seq4, and D5 seq5 variants were descendants of the D5 seq1 (D5-Okinawa) variant (Figure 1A). In MeaNS (WHO Measles Nucleotide Surveillance Database) (WHO and PHE) 222 D5 strains to an identical N450 sequence with D5-Okinawa strain are reported (as of December 22, 2018). They were distributed globally and detected in the United States, Canada, many European countries, Taiwan, Korea, and Japan between 2007 and 2009. However, the genotype D5 strains, including the D5-Okinawa strain, have not been reported in Japan and also globally after 2010 (World Health Organization [WHO], 2015).

### Genotype D4

Genotype D4 strains detected in Japan could be divided into six different sequence variants (D4 seq1 to D4 seq6 [D4 seq1–6] variants). Phylogenetic analysis was performed using a dataset consisted of the D4 seq1–6 variants, WHO D4 reference strain (MVi/Montreal.CAN/0.89 [D4]), and 11 WHO-named strains. In addition, one D4 strain detected outside of Japan (overseas D4 strain) with the identical N450 sequence to the D4 seq5 variant was included (Figure 1B). The tree was constructed together with genotype D9 strains (Figure 1B). The majority (*n* = 58, 89%) was D4 seq3 variant, which had an identical N450 sequence to the MVs/Manchester.GBR/10.09/ named strain (D4-Manchester). The D4-Manchester strain was first detected in United Kingdom in 2009 and caused outbreaks across the central and continental western Europe between 2010 and 2013 (Pfaff et al., 2010; Fitzpatrick et al., 2012; Necula et al., 2013; O’Riordan et al., 2014; Santibanez et al., 2015). D4 seq1 variant with an identical N450 sequence to the MVs/Enfield.GBR/14.07/ named strain (D4-Enfield) was detected from three cases (one in 2008 and two in 2011). The D4-Enfield strain was first detected in United Kingdom in 2007 and circulated endemically in the United Kingdom in 2008 (O’Riordan et al., 2014; Santibanez et al., 2015). The D4-Enfield strain has also caused outbreaks in other European countries (Rogalska et al., 2010; D’Agaro et al., 2011). D4 seq6 variant with an identical N450 sequence to the MVs/Marmande.FRA/43.11/2 named strain (D4-Marmande) was detected from one case in 2012. The D4-Marmande strain was first detected in France in 2011.

### Genotype D9

In total, 108 genotype D9 strains were detected between 2009 and 2015. These strains were divided into as many as 19 different sequence variants (D9 seq1 to D9 seq19 [D9 seq1–19] variants). A phylogenetic analysis was performed using a dataset consisted of the D9 seq1–19 variants, WHO D9 reference strain (MVi/Victoria.AUS/12.99 [D9]), and two WHO-named strains. In addition, 11 overseas D9 strains with the identical sequences to one or other D9 seq1–19 variants were included (Figure 1B). The tree was constructed together with genotype D4 strains. Unlike the genotype D4 strains, the majority of the genotype D9 strains had no matched named strain with the identical N450 sequence. Exceptionally, D9 seq12 variant had an identical N450 sequence to the MVs/Bristol.GBR/13.05/ (D9-Bristol)-named strain. Between 2011 and 2012, D9-Bristol strain was detected in many cases in Malaysia and Turkey, or from individuals with travel history to Malaysia and Indonesia (data from MeaNS; WHO and PHE). In Japan, the D9 seq12 (D9-Bristol) variant was detected from eight cases (one, three, and four in 2011, 2012, and 2013, respectively). The D9 seq15 variant detected in 2012 in Japan was designated as a named strain (MVs/Yamanashi.JPN/51.12/ [D9-Yamanashi]). The D9 seq15 (D9-Yamanashi) variant was also detected in four cases in 2014 and an additional four cases in 2015. However, the D9 seq15 (D9-Yamanashi) variant was not considered to be endemic in Japan between 2012 and 2015 because the index case of each D9 seq15 (D9-Yamanashi) outbreak in 2014 and 2015 had a travel history to Indonesia or Malaysia (National Institute of Infetious Diseases [NIID], and Tuberculosis, and Infectious Disease Control Division, Ministry of Health, Labor, and Welfare [MHLW], 2015a,b, 2016a,b; World Health Organization [WHO] and Public Health England (PHE), 2019). In Indonesia, the genotype D9 and G3 strains were endemically circulated (D8 was newly detected in 2014) (Subangkit et al., 2017). In Malaysia, both genotype D9 and D8 strains were predominantly detected between 2013 and 2016 (Hagan et al., 2018). Between 2013 and 2015, D9-Yamanashi strain was also detected in Australia, Belarus, Germany, and United States from individuals with travel history to Indonesia or Malaysia (WHO and PHE).

### Genotype H1

**FIGURE 2:**
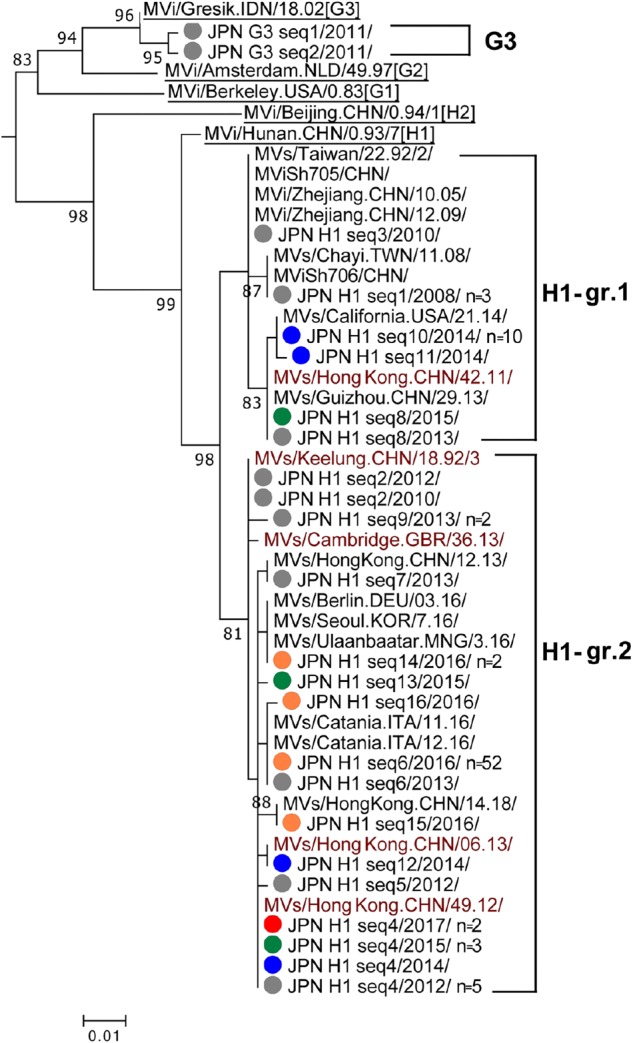
Phylogenetic tree of the genotype G3 and H1 MV strains. The analysis and labeling methods of MV strains are described in the legend of Figure 1. Two lineages in H1 genotype (H1-gr.1 and H1-gr.2) tentatively proposed in this study are indicated.

Genotype H1 strains detected in Japan consisted of 16 different sequence variants (H1 seq1 to H1 seq16 [H1 seq1–16] variants). A phylogenetic analysis was performed using a dataset consisting of the H1 seq1–16 variants, WHO H1 reference strain (MVi/Hunan.CHN/0.93/7 [H1]), and five WHO-named strains. In addition, 15 overseas H1 strains with the identical N450 sequences to one or another H1 seq1–16 variants were included (Figure 2). The tree was constructed together with the WHO H2 reference strain (MVi/Beijing.CHN/0.94/1 [H2]), G1 reference strain (MVi/Berkeley.USA/0.83 [G1]), and genotype G3 strains (Figure 2). H1 seq1–16 variants were clustered into two distinct groups (H1-gr.1 and H1-gr.2). H1-gr.1 included the MVs/Hong Kong.CHN/42.11/ (H1-HK11) named strain. The H1 seq8 variant had the identical N450 sequence to the H1-HK11 named strain. H1-gr.2 included four WHO-named strains, MVs/Keelung.CHN/18.92/3 (H1-Keelung), MVs/Cambridge.GBR/36.13/, MVs/Hong Kong.CHN/06.13/(H1-HK13), and MVs/Hong Kong.CHN/49.12/(H1-HK12). H1 seq2, H1 seq4, and H1 seq12 variants had the identical sequences to H1-Keelung, H1-HK12, and H1-HK13 named strains, respectively. Among the 16 H1 different sequence variants, four variants were detected in multiple years: i.e., the H1 seq8 variant was detected in 2013 and 2015, while the H1 seq2 variant was detected in 2010 and 2012, and H1 seq6 variant was detected in 2013 and disappeared between 2014 and 2015. However, this strain was imported again into Japan in 2016 through the Kansai International Airport and caused an outbreak (Watanabe et al., 2017; Kaida et al., 2018). As a result, the H1 seq6 variant was detected from 52 cases in 2016 (Figure 2). The possible index case of this outbreak started at the Kansai International Airport was a returnee from China to Japan (Watanabe et al., 2017). The H1 seq4 (H1-HK12) variant was most frequently detected in Japan. It was detected in 2012, 2014, 2015, and 2017. The H1-HK12 strains were the most predominant strains in China and >4,000 strains are reported in the MeaNS database (WHO and PHE) (as of December 15, 2018).

### Genotype G3

The two genotype G3 strains detected in Japan in 2011 (G3 seq1 and G3 seq2 [G3 seq1–2] variants) (Figure 2) had an epidemiological linkage to Indonesia (Tanaka et al., 2011; National Institute of Infetious Diseases [NIID], and Tuberculosis, and Infectious Disease Control Division, Ministry of Health, Labor, and Welfare [MHLW], 2012a,b). Indeed, the G3 genotype strains have been an endemic genotype in Indonesia (Rota et al., 2011; Subangkit et al., 2017).

### Genotype B3

**FIGURE 3:**
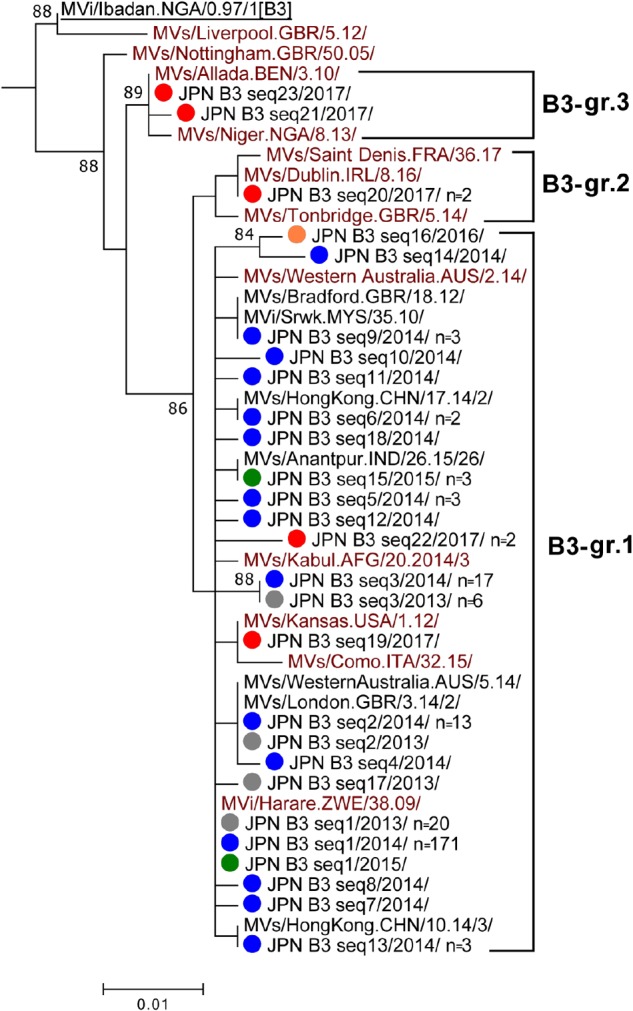
Phylogenetic tree of the genotype B3 MV strains. The analysis and labeling methods of MV strains are described in the legend of Figure 1. Three lineages (B3-gr.1, B3-gr.2, and B3-gr.3) tentatively proposed in this study are indicated.

Genotype B3 strains detected in Japan consisted of 23 different sequence variants (B3 seq1 to B3 seq23 variants [B3 seq1–23]) and largely classified into three groups (B3-gr.1, B3-gr.2, and B3-gr.3) (Figure 3). The majority of genotype B3 strains in Japan belonged to B3-gr.1, which includes five named strains, MVi/Harare.ZWE/38.09/ (B3-Harare), MVs/Kansas.USA/1.12/ (B3-Kansas), MVs/Como.ITA/32.15/, MVs/Kabul.AFG/20.2014/3, and MVs/Western Australia.AUS/2.14/. The B3 seq1 and B3 seq19 variants had the identical N450 sequences to the B3-Harera and B3-Kansas named strains, respectively (Figure 3). The B3 seq19 variant (B3-Kansas) was detected from only a single case, while B3 seq1 variant (B3-Harare) was most frequently detected in Japan (from 20 cases in 2013 and 171 cases in 2014) (Table 1). Thus, B3 seq1 variant (B3-Harare), which was repeatedly imported from Philippines (Takahashi et al., 2014; Minagawa et al., 2015; Miyoshi et al., 2015, 2017; National Institute of Infetious Diseases [NIID], and Tuberculosis, and Infectious Disease Control Division, Ministry of Health, Labor, and Welfare [MHLW], 2015a,b; Yagi et al., 2015; Kaida et al., 2018), was the major cause of measles outbreaks in 2013 and 2014 in Japan. Between 2013 and 2016, outbreaks by the B3-Harare strain have been also observed in European countries (Rasmussen et al., 2015; Magurano et al., 2016, 2017; Santibanez et al., 2017). Data from the MeaNS sequence database (WHO and PHE) show that B3-Harare strain has been imported into many other countries from the Philippines and detected worldwide between 2013 and 2015. Transmission of the B3-Harare strain was interrupted in Japan and no B3-Harare strain was detected after 2016.

B3-gr.2 contains three named strains: i.e., MVs/Saint Denis.FRA/36/17, MVs/Dublin.IRL/8.16/ (B3-Dublin), and MVs/Tonbridge.GBR/5.14/. In 2017, the B3 seq20 variant with an identical N450 sequence to the B3-Dublin named strain was detected from two cases in Japan (Figure 3). The B3-Dublin strain circulated in many European countries after 2016 (WHO and PHE; Amendola et al., 2017; Santibanez et al., 2017) and the two cases in Japan had travel history to Italy. B3-gr.3 contains two named strains: i.e., MVs/Allada.BEN/3.10/ (B3-Allada) and MVs/Niger.NGA/8.13/. MV strains in B3-gr.3 (B3 seq21 and B3 seq23 variants) were detected from two cases in 2017 in Japan. B3 seq23 variant had an identical N450 sequence to the B3-Allada named strain (Figure 3). After 2016, the B3-Allada strain was detected only in Spain, United Kingdom, and Nigeria (one case each from these countries) (WHO and PHE).

### Genotype D8

**FIGURE 4:**
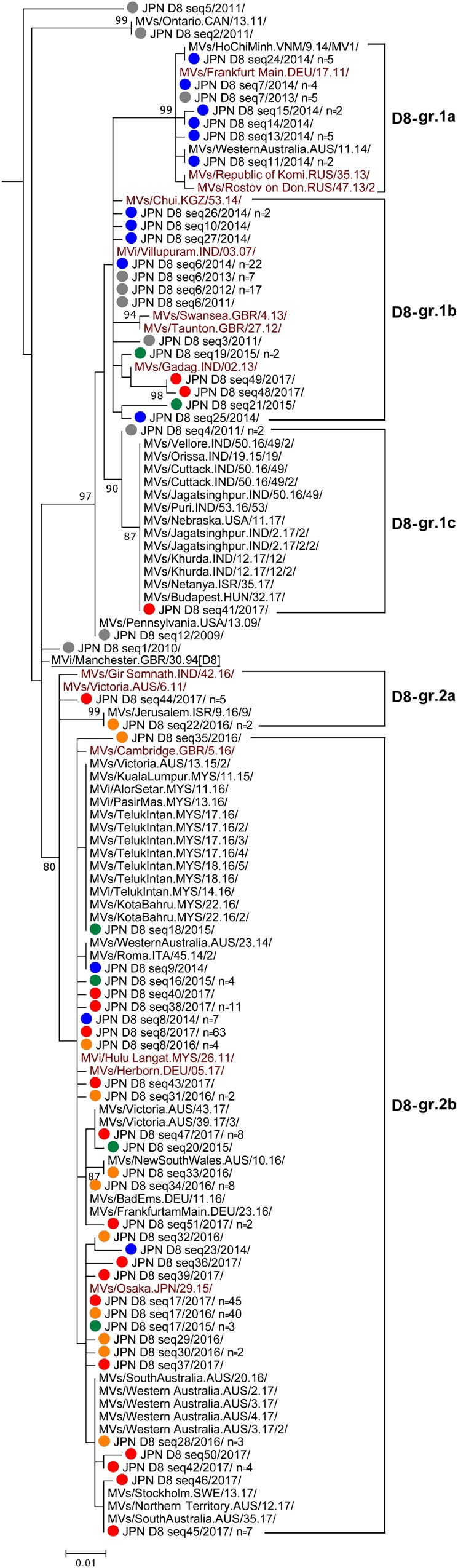
Phylogenetic tree of the genotype D8 MV strains. The analysis and labeling methods of MV strains are described in the legend of Figure 1. Five lineages (D8-gr.1a, D8-gr.1b, D8-gr.1c, D8-gr.2a, and D8-gr.2b) tentatively proposed in this study are indicated.

Genotype D8 strains detected in Japan between 2009 and 2017 were divided into as many as 51 different sequence variants (D8 seq1 to D8 seq51 [D8 seq1–51] variants). A phylogenetic analysis was performed using a dataset consisted of the D8 seq1–51 variants, WHO D8 reference strain (MVi/Manchester.GBR/30.94 [D8]), and 14 WHO-named strains (Figure 4). In addition, 46 overseas D8 strains with the identical sequences to one or another D8 seq1–51 variants were included (Figure 4). The majority of the D8 seq1–51 variants were largely classified into two groups (D8-gr.1 and D8-gr.2). In the present study, D8-gr.1 was further classified into three sub-groups (D8-gr.1a, D8-gr.1b, and D8-gr.1c), and D8-gr.2 was classified into two sub-groups (D8-gr.2a and D8-gr.2b) (Figure 4).

In 2014, D8-gr.1a, D8-gr.1b, and D8-gr.2b MV strains were detected in Japan. D8-gr.1a, D8-gr.1b, and D8-gr.2b contain three, five, and four WHO-named strains, respectively, suggesting that MV strains in these sub-groups circulated globally. The D8 seq6 variant in D8-gr.1b was one of the major strains detected in Japan between 2011 and 2014. The D8 seq6 variant had the identical N450 sequence to the MVi/Villupuram.IND/03.07/ (D8-Villupuram) named strain. The D8-Villupuram strain has been detected worldwide between 2010 and 2015 (WHO and PHE). In 2015, only 35 measles cases were detected in Japan, and 12 measles cases were caused by genotype D8 strains in the D8-gr.1b and D8-gr.2b (Figure 4 and Table 1). In 2016, the D8-gr.2a MV strains were detected for the first time in Japan together with the D8-gr.2b MV strains. No D8-gr.1 MV strains were detected in 2016, suggesting interruption of the transmission of D8-gr.1 MV strains. In 2017, the D8-gr.1b MV strains were again detected together with D8-gr.2a and D8-gr.2b MV strains. In 2017, the D8-gr.1c MV strains were detected for the first time in Japan from a single case.

Between 2015 and 2017, the D8 seq17 variant in D8-gr.2b was most frequently detected in Japan (*n* = 3, 40, and 45 in 2015, 2016, and 2017, respectively). The D8 seq17 variant detected in Japan was designated as a WHO-named strain (MVs/Osaka.JPN/29.15/ [D8-Osaka]). D8-Osaka strain was detected worldwide between 2016 and 2018 (WHO and PHE; Amendola et al., 2017). Many imported D8-Osaka cases from Indonesia and Thailand were reported in Japan, Australia, United States, and European countries between 2015 and 2018 (WHO and PHE). The D8 seq8 variant in D8-gr.2b was the second most frequently detected strain in Japan. The D8 seq8 variant had an identical N450 sequence to the MVi/Hulu Langat.MYS/26.11/ (D8-HL) named strain. The D8 seq8 (D8-HL) variant was detected from 7, 4, and 63 cases in 2014, 2016, and 2017, respectively, in Japan (Figure 4). Between 2014 and 2017, the D8-HL strain was detected worldwide, including in Indonesia (WHO and PHE).

### Chronological Analysis of MV Strains

**FIGURE 5:**
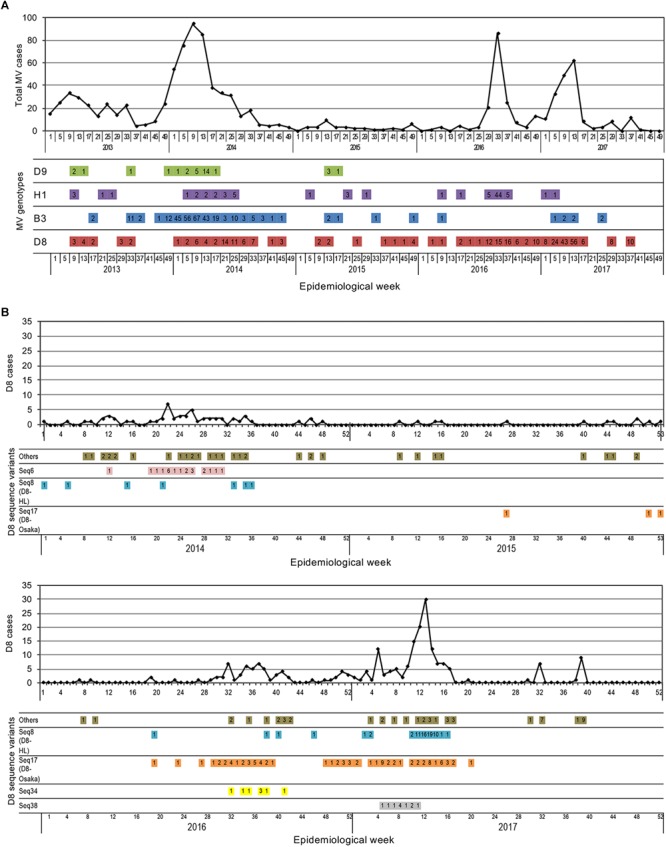
Time course of detection of MV strains by week. **(A)** The time course detection of all genotype strains detected between 2013 and 2017. The line graphs show the total number of reported measles cases. The colored horizontal bars show the genotype data of MV strains. The number of reported measles cases for each genotype is shown in the colored horizontal bars. **(B)** The time course detection of genotype D8 strains detected between 2014 and 2017. The line graphs show the total number of reported genotype D8 MV cases. The colored horizontal bars show the data of individual sequence variant D8 MV strains. The number of reported measles cases for each sequence variant D8 MV strain is shown in the colored horizontal bars.

On March 27, 2015, the RVC for measles elimination in WPR verified that Japan achieved measles elimination. However, when epidemiological or laboratory evidence indicates the presence of a chain of transmission of an MV strain that persists for >12 months in a defined geographical area, it is considered that reestablishment of endemic transmission has occurred in the area where measles had been eliminated previously (World Health Organization [WHO], 2013b). To understand the situation of MV transmission in Japan in the postelimination era, the detection pattern of each MV sequence variant was analyzed in chronological order. The 891 MV strains detected between 2008 and 2017 were classified into seven genotypes (D5, D4, D9, H1, G3, B3, and D8) and 124 sequence variants (D5 seq1–7, D4 seq1–6, D9 seq1–19, H1 seq1–16, G3 seq1–2, B3 seq1–23, and D8 seq1–51). Among them, the strains detected in the postelimination era (between 2015 and 2017) were classified into 48 MV sequence variants (1, 7, 8, and 32 sequence variants in the D9, H1, B3, and D8 genotypes, respectively). Among the 48 MV sequence variants, most variants were detected in periods shorter than 5 weeks (Supplementary Tables 2–5). Four MV sequence variants (D9 seq15, H1 seq4, H1 seq6, D8 seq38, and D8 seq34) were detected in a period of 5–10 weeks. Only two MV sequence variants, D8 seq8 and D8 seq17, were detected for periods longer than 10 weeks (Supplementary Table 5). More importantly, these variants (D8 seq8 and D8 seq17) were detected for a consecutive 2 and 3 years, respectively (Figure 5 and Supplementary Table 5). The line graphs in Figure 5 show the number of reported measles cases by week (data from Infectious Agents Surveillance Report, Infectious Disease Surveillance Center, NIID, Japan), and the colored horizontal bars in Figure 5 show the genotype data of MV strains. The ratio of measles cases with genotype information was low in 2013, but most measles cases after 2014 included MV genotype data. It was clear that the genotype D9, H1, and B3 strains were detected only intermittently with long (>4 weeks) nondetection periods. However, the genotype D8 strains were detected constantly even during the postelimination era between 2015 and 2017 (Figure 5A).

When the D8 seq1–51 variants were assessed individually, most of them were detected only sporadically (Supplementary Table 5). Certain strains caused small outbreaks, but were detected only for a short period. Only two sequence variants (D8 seq8 [D8-HL] and D8 seq17 [D8-Osaka]) were detected many times over 2 or 3 years, respectively (Figure 5B and Supplementary Table 5). Figure 5B shows the detection data of genotype D8 strains by week. The D8 seq8 (D8-HL) variants were detected in the 19th, 38th, 40th, and 46th weeks in 2016 and the 2nd, 3rd, and consecutive 7 weeks between the 10th and 16th weeks. Since each sporadic case or small outbreak was separated by a long period (more than 4 weeks), the transmission of D8 seq8 (D8-HL) variants has not persisted for >12 months in Japan. In 2015, the D8 seq17 (D8-Osaka) variants were only sporadically detected in the 27th, 51th, and 53th weeks (Figure 5B). Between 2016 and 2017, the D8 seq17 (D8-Osaka) variants were detected for a long period of time (from the 19th week in 2016 until the 20th week in 2017). During this period, in total, D8 seq17 variant (D8-Osaka) was detected in 85 MV cases, including those in 11 small outbreaks (*n* = 2–16). However, the detection period was apparently separated into two periods. The former period started from 19th week in 2016 and ended at 39th week in the same year, while the latter period started from the 48th week in 2016 and ended at the 20th week in 2017. Between these two periods, there was an 8-week interval when no D8 seq17 (D8-Osaka) variant was detected (Figure 5B and Supplementary Table 5). In addition, the geographic distribution of the measles cases in the two periods was separated. The measles cases in the former period were detected in three prefectures in the Kansai region, a western region of Japan, and the measles cases in the latter period were detected in two prefectures in the Kanto region, in an eastern region of Japan. D8 seq17 (D8-Osaka) variant was not detected after the 21th week in 2017 (as of December 15, 2018). These data suggested that transmission of D8 seq17 (D8-Osaka) variant did not persist for >12 months in Japan.

## Conclusion

In this study, we analyzed the molecular epidemiology of MV strains between 2008 and 2017 in Japan. The genotyping evidence that supports the interruption of endemic MV transmission is one of the essential criteria to be verified as achieving measles elimination. Thus, MV detection testing by RT-PCR and genotype analysis has been performed on most of the measles cases in PHIs nationwide in Japan. Following these efforts, Japan had strong molecular epidemiological evidence that supports the interruption of endemic MV transmission in Japan. Accordingly, Japan was successfully verified to have achieved measles elimination in March 2015. This effort to detect MV by PCR and genotyping has continued during the elimination era in Japan. The molecular epidemiological data of MV in certain prefectures or cities were reported in previous studies (Aoki et al., 2009; Kurata et al., 2009; Nagano et al., 2011; Tanaka et al., 2011; Watanabe et al., 2012; Miyoshi et al., 2014, 2015, 2017; Nadaoka et al., 2014; Minagawa et al., 2015; Yagi et al., 2015; Kaida et al., 2018; Komabayashi et al., 2018), but a nationwide analysis was conducted in this study for the first time. The data demonstrated that many different MV strains were detected in Japan. Although certain genotypes have been detected almost every year, the detailed sequence analysis of individual MV strains revealed multiple importations and disappearance of great numbers of different MV strains. Epidemiological investigations have also detected overseas travel histories in many measles cases. Especially, WHO-named strains were detected repeatedly in recent years, because these named strains have circulated in many countries worldwide. Demonstrating the elimination status in the situation with repeated importation of the same strains is challenging. Nevertheless, the detailed sequence data of individual MV strains and chronological analysis of these strains provided sufficient evidence showing that Japan has still maintained its measles elimination status.

## Data Availability

The datasets generated for this study can be found in GenBank, All accession numbers are listed in Supplementary Table 1.

## Ethics Statement

The Infectious Diseases Control Law in Japan defines measles as a notifiable infectious disease and the MHLW Measles Guideline under the Infectious Diseases Control Law requests mandatory reporting of all measles cases. The Guideline also requests for physicians and local governments to conduct MV detection test by PCR and genotype analysis for all measles suspected cases in principle. The Ethics Committee of the NIID agreed to analyze and publish the MV epidemiological data in academic journals (No. 975).

## Author Contributions

FS, MT, and KS designed the study. FS, MM, TI, HN, MS, MI, HM, TKu, RO, JK, TKa, KKom, KT-T, TS, KO, NOk, HK, SS, KKoz, NOt, YM, KS, and MT analyzed the data. FS, MT, and KS wrote the manuscript. All authors reviewed the manuscript.

## Conflict of Interest Statement

The authors declare that the research was conducted in the absence of any commercial or financial relationships that could be construed as a potential conflict of interest.

## Footnotes

1 http://www.who-measles.org/Public/Web_Front/main.php

## Abbreviations

MV: measles virus
MHLW: Ministry of Health, Labour and Welfare, Japan
PCR: polymerase chain reaction
PHIs: public health institutes
WHO: World Health Organization

## References

[B1] Amendola A. Bianchi S. Frati E. R. Ciceri G. Faccini M. Senatore S. (2017). Ongoing large measles outbreak with nosocomial transmission in Milan, northern Italy, March-August 2017. *Euro. Surveill.* 22:30596. 10.2807/1560-7917.ES.2017.22.33.30596 28840825 PMC5572939

[B2] Aoki Y. Mizuta K. Suto A. Ikeda T. Abiko C. Yamaguchi I. (2009). Importation of the evolving measles virus genotype d9 to yamagata, Japan from Thailand in 2009. *Jpn. J. Infect. Dis.* 62 481–482. 19934546

[B3] Centeno R. Fuji N. Okamoto M. Dapat C. Saito M. Tandoc A. (2015). Genetic characterization of measles virus in the Philippines, 2008-2011. *BMC Res. Notes* 8:211. 10.1186/s13104-015-1201-1 26036942 PMC4467837

[B4] Cheng W. Y. Tung H. P. Wang H. C. Lee L. L. Wu H. S. Liu M. T. (2013). Molecular epidemiology of measles virus in Taiwan in 2010-2011: the common genotype changed from H1 to D9 and the first appearance of D4. *J. Med. Virol.* 85 1095–1099. 10.1002/jmv.23563 23588738

[B5] Dabbagh A. Laws R. L. Steulet C. Dumolard L. Mulders M. N. Kretsinger K. (2018). Progress Toward Regional Measles Elimination - Worldwide, 2000-2017. *MMWR Morb. Mortal Wkly. Rep.* 67 1323–1329. 10.15585/mmwr.mm6747a6 30496160 PMC6276384

[B6] D’Agaro P. Molin G. D. Gallo T. Rossi T. Santon D. Busetti M. (2011). Epidemiological and molecular assessment of a measles outbreak in a highly vaccinated population of northeast Italy. *Epidemiol. Infect.* 139 1727–1733. 10.1017/S095026881100032X 21396148

[B7] Fitzpatrick G. Ward M. Ennis O. Johnson H. Cotter S. Carr M. J. (2012). Use of a geographic information system to map cases of measles in real-time during an outbreak in Dublin, Ireland, 2011. *Euro. Surveill.* 17:20330. 23231894 10.2807/ese.17.49.20330-en

[B8] Fuji N. Suzuki A. Saito M. Centeno R. Galang H. Lupisan S. (2011). Interruption of the circulation of an indigenous measles genotype and the introduction of other genotypes after a mass vaccination campaign in the Philippines. *J. Med. Virol.* 83 1424–1427. 10.1002/jmv.22103 21618549 PMC3378693

[B9] Hagan J. E. Kriss J. L. Takashima Y. Mariano K. M. L. Pastore R. Grabovac V. (2018). Progress Toward Measles Elimination - Western Pacific Region, 2013-2017. *MMWR Morb. Mortal Wkly Rep.* 67 491–495. 10.15585/mmwr.mm6717a3 29723171 PMC5933871

[B10] Ho H. J. Low C. Ang L. W. Cutter J. L. Tay J. Chan K. P. (2014). Progress towards measles elimination in Singapore. *Vaccine* 32 6927–6933. 10.1016/j.vaccine.2014.10.046 25444818

[B11] Jayamaha J. Binns P. L. Fennell M. Ferson M. J. Newton P. Tran T. (2012). Laboratory diagnosis, molecular characteristics, epidemiological and clinical features of an outbreak of measles in a low incidence population in Australia. *J. Clin. Virol.* 54 168–173. 10.1016/j.jcv.2012.02.025 22459002

[B12] Kaida A. Iritani N. Kanbayashi D. Yamamoto S. P. Hirai Y. Hakui N. (2018). Ten-year surveillance of measles virus from 2007-2016 in Osaka City, Japan. *Jpn. J. Infect. Dis.* 71 152–154. 10.7883/yoken.JJID.2017.322 29279450

[B13] Komabayashi K. Seto J. Tanaka S. Suzuki Y. Ikeda T. Onuki N. (2018). The largest measles outbreak, including 38 modified measles and 22 typical measles cases in its elimination era in Yamagata, Japan, 2017. *Jpn. J. Infect. Dis.* 71 413–418. 10.7883/yoken.JJID.2018.083 29962488

[B14] Kurata T. Miyagawa H. Furutani E. Kase T. Takahashi K. (2009). An outbreak of measles classified as genotype H1 in 2008 in Osaka Prefecture. *Jpn. J. Infect. Dis.* 62 76–77. 19168968

[B15] Lo N. C. Hotez P. J. (2017). Public health and economic consequences of vaccine hesitancy for measles in the United States. *JAMA Pediatr.* 171 887–892. 10.1001/jamapediatrics.2017.1695 28738137 PMC5710408

[B16] Magurano F. Baggieri M. Bordi L. Lalle E. Chironna M. Lazzarotto T. (2016). Measles in Italy: co-circulation of B3 variants during 2014. *J. Med. Virol.* 88 1081–1085. 10.1002/jmv.24416 26496509

[B17] Magurano F. Baggieri M. Filia A. Del Manso M. Lazzarotto T. Amendola A. (2017). Towards measles elimination in Italy: virological surveillance and genotypes trend (2013-2015). *Virus Res.* 236 24–29. 10.1016/j.virusres.2017.05.009 28522332

[B18] Minagawa H. Yasui Y. Adachi H. Ito M. Hirose E. Nakamura N. (2015). Case-based surveillance enhanced with measles virus detection/genotyping is essential to maintain measles elimination in Aichi Prefecture. *Jpn. Vaccine* 33 6043–6048. 10.1016/j.vaccine.2015.08.070 26342850

[B19] Miyoshi M. Komagome R. Ishida S. Kikuchi M. Sato H. Ito H. (2014). Recent progress toward measles elimination in Hokkaido, Japan, during 2011-2012. *Jpn. J. Infect. Dis.* 67 311–314. 10.7883/yoken.67.311 25056081

[B20] Miyoshi M. Komagome R. Ishida S. Ohnishi A. Furudate T. Mizushima Y. (2015). Import-associated measles outbreak including hospital- and clinic-based transmission in the non-Endemic Hokkaido District, Japan, 2014. *Jpn. J. Infect. Dis.* 68 451–453. 10.7883/yoken.jjid.2015.237 26399929

[B21] Miyoshi M. Komagome R. Yamaguchi H. Ohnishi A. Kikuchi M. Ishida S. (2017). Detection of measles virus genotypes B3, D4, D5, D8, and H1 in the surveillance system in Hokkaido, Japan, 2006-2015, the last decade toward the elimination. *Jpn. J. Infect. Dis.* 70 317–319. 10.7883/yoken.JJID.2016.253 28003595

[B22] Morita Y. Suzuki T. Shiono M. Shiobara M. Saitoh M. Tsukagoshi H. (2007). Sequence and phylogenetic analysis of the nucleoprotein (N) gene in measles viruses prevalent in Gunma, Japan, in 2007. *Jpn. J. Infect. Dis.* 60 402–404. 18032846

[B23] Nadaoka Y. Hayata N. Sugishita Y. Kajiwara T. Watanabe Y. Yoshida M. (2014). The 2011 measles outbreak in Tokyo. An analysis of surveillance data. *Nihon Koshu Eisei Zasshi* 61 136–144. 24739941

[B24] Nagai M. Xin J. Y. Yoshida N. Miyata A. Fujino M. Ihara T. (2009). Modified adult measles in outbreaks in Japan, 2007-2008. *J. Med. Virol.* 81 1094–1101. 10.1002/jmv.21372 19382253

[B25] Nagano H. Jinushi M. Komagome R. Miyoshi M. Kikuchi M. Muratsubaki E. (2011). Progress toward measles elimination between 2008 and 2010 in the Hokkaido district. Japan. *Jpn. J. Infect. Dis.* 64 445–447. 21937832

[B26] National Institute of Infetious Diseases [NIID], and Tuberculosis and Infectious Disease Control Division, Ministry of Health, Labor and Welfare [MHLW] (2011a). *Measles in Japan.* Available: http://idsc.nih.go.jp/iasr/32/372/tpc372.html (accessed February 2011).

[B27] National Institute of Infetious Diseases [NIID], and Tuberculosis and Infectious Disease Control Division, Ministry of Health, Labor and Welfare [MHLW] (2011b). *Measles in Japan, 2010.* Luton: IASR.

[B28] National Institute of Infetious Diseases [NIID], and Tuberculosis, and Infectious Disease Control Division, Ministry of Health, Labor, and Welfare [MHLW] (2012a). *Measles in Japan, 2011.* Luton: IASR.

[B29] National Institute of Infetious Diseases [NIID], and Tuberculosis, and Infectious Disease Control Division, Ministry of Health, Labor, and Welfare [MHLW] (2012b). *Measles in Japan, 2011.* Available: http://idsc.nih.go.jp/iasr/33/384/tpc384.html (accessed May 2015).

[B30] National Institute of Infetious Diseases [NIID], and Tuberculosis and Infectious Disease Control Division, Ministry of Health, Labor and Welfare [MHLW] (2013a). *Measles in Japan, 2012.* Available: http://www.niid.go.jp/niid/en/iasr-measles-e/865-iasr/4238-tpc396.html (accessed February 2013).

[B31] National Institute of Infetious Diseases [NIID], and Tuberculosis and Infectious Disease Control Division, Ministry of Health, Labor and Welfare [MHLW] (2013b). *Measles in Japan, 2012.* Luton: IASR.

[B32] National Institute of Infetious Diseases [NIID], and Tuberculosis, and Infectious Disease Control Division, Ministry of Health, Labor, and Welfare [MHLW] (2015a). Measles in Japan, as of March 2015. *IASR* 36 51–53.

[B33] National Institute of Infetious Diseases [NIID], and Tuberculosis, and Infectious Disease Control Division, Ministry of Health, Labor, and Welfare [MHLW] (2015b). *Measles in Japan, as of March 2015.* Available: https://www.niid.go.jp/niid/en/iasr-measles-e/865-iasr/5592-tpc422.html (accessed April 2015).

[B34] National Institute of Infetious Diseases [NIID], and Tuberculosis, and Infectious Disease Control Division, Ministry of Health, Labor, and Welfare [MHLW] (2016a). Measles, and rubella/congenital rubella symdrome in Japan. as of March 2016. *IASR* 37 59–61.

[B35] National Institute of Infetious Diseases [NIID], and Tuberculosis, and Infectious Disease Control Division, Ministry of Health, Labor, and Welfare [MHLW] (2016b). *Measles and rubella/congenital rubella symdrome in Japan, as of March 2016.* Available: https://www.niid.go.jp/niid/en/iasr-vol33-e/865-iasr/6460-434te.html (accessed April 2016).

[B36] National Institute of Infetious Diseases [NIID], and Tuberculosis and Infectious Disease Control Division, Ministry of Health, Labor and Welfare [MHLW] (2017a). *Measles in Japan, 2016.* Luton: IASR.

[B37] National Institute of Infetious Diseases [NIID], and Tuberculosis and Infectious Disease Control Division, Ministry of Health, Labor and Welfare [MHLW] (2017b). Measles in Japan, 2016. *IASR* 38 45–47.

[B38] National Institute of Infetious Diseases [NIID], and Tuberculosis and Infectious Disease Control Division, Ministry of Health, Labor and Welfare [MHLW] (2018a). *Measles in Japan, as of February 2018.* Luton: IASR.

[B39] National Institute of Infetious Diseases [NIID], and Tuberculosis and Infectious Disease Control Division, Ministry of Health, Labor and Welfare [MHLW] (2018b). Measles in Japan, as of February 2018. *IASR* 39 49–51.

[B40] Necula G. Lazar M. Stanescu A. Pistol A. Santibanez S. Mankertz A. (2013). Transmission and molecular characterisation of wild measles virus in Romania, 2008 to 2012. *Euro. Surveill.* 18:20658. 10.2807/1560-7917.es2013.18.50.20658 24342518

[B41] Nic Lochlainn L. Mandal S. De Sousa R. Paranthaman K. Van Binnendijk R. Ramsay M. (2016). A unique measles B3 cluster in the United Kingdom and the Netherlands linked to air travel and transit at a large international airport, February to April 2014. *Euro. Surveill.* 21:30177. 10.2807/1560-7917.ES.2016.21.13.30177 27074646

[B42] Okafuji T. Okafuji T. Fujino M. Nakayama T. (2006). Current status of measles in Japan: molecular and seroepidemiological studies. *J. Infect. Chemother.* 12 343–348. 10.1016/s1341-321x(06)70892-3 17235638

[B43] O’Riordan B. Carr M. J. Connell J. Dunford L. Hall W. W. Hassan J. (2014). Seroepidemiology and phylogenetic characterisation of measles virus in Ireland, 2004-2013. *J. Clin. Virol.* 60 374–380. 10.1016/j.jcv.2014.05.010 24929750

[B44] Park S. H. Lee D. H. Jin J. Y. Shin Y. L. Shin M. Kim S. S. (2017). Measles outbreaks in the Kyeongin area of the Republic of Korea, 2013-2014: A single-center experience in a country of measles elimination. *Asian Pac. J. Trop. Med.* 10 69–74. 10.1016/j.apjtm.2016.12.003 28107869

[B45] Park Y. J. Eom H. S. Kim E. S. Choe Y. J. Bae G. R. Lee D. H. (2013). Reemergence of measles in South Korea: implications for immunization and surveillance programs. *Jpn. J. Infect. Dis.* 66 6–10. 10.7883/yoken.66.6 23429077

[B46] Pfaff G. Lohr D. Santibanez S. Mankertz A. Van Treeck U. Schonberger K. (2010). Spotlight on measles 2010: Measles outbreak among travellers returning from a mass gathering, Germany, September to October 2010. *Euro. Surveill.* 15:19750. 21172175

[B47] Pham V. H. Nguyet D. P. Mai K. N. Truong K. H. Huynh L. V. Pham T. H. (2014). Measles epidemics among children in vietnam: genomic characterization of virus responsible for measles outbreak in Ho Chi Minh City, 2014. *EBioMedicine* 1 133–140. 10.1016/j.ebiom.2014.10.015 26137521 PMC4457408

[B48] Rasmussen L. D. Fonager J. Knudsen L. K. Andersen P. H. Ronn J. Poulsen M. W. (2015). Phylogenetic and epidemiological analysis of measles outbreaks in Denmark, 2013 to 2014. *Euro. Surveill.* 20:30027. 10.2807/1560-7917.ES.2015.20.39.30027 26537105

[B49] Rogalska J. Santibanez S. Mankertz A. Makowka A. Szenborn L. Stefanoff P. (2010). Spotlight on measles 2010: An epidemiological overview of measles outbreaks in Poland in relation to the measles elimination goal. *Euro. Surveill.* 15:19549. 20460084 10.2807/ese.15.17.19549-en

[B50] Rosewell A. Reinten-Reynolds T. Spokes P. J. (2012). EpiReview: Measles in NSW, 2002-2011. *N S W Publ. Health Bull.* 23 201–207. 10.1071/NB12085 23442997

[B51] Rota P. A. Brown K. Mankertz A. Santibanez S. Shulga S. Muller C. P. (2011). Global distribution of measles genotypes and measles molecular epidemiology. *J. Infect Dis.* 204(Suppl. 1) S514–S523. 10.1093/infdis/jir118 21666208

[B52] Santibanez S. Hubschen J. M. Ben Mamou M. C. Muscat M. Brown K. E. Myers R. (2017). Molecular surveillance of measles and rubella in the WHO European Region: new challenges in the elimination phase. *Clin. Microbiol. Infect.* 23 516–523. 10.1016/j.cmi.2017.06.030 28712666

[B53] Santibanez S. Hubschen J. M. Muller C. P. Freymuth F. Mosquera M. M. Mamou M. B. (2015). Long-term transmission of measles virus in Central and continental Western Europe. *Virus Genes* 50 2–11. 10.1007/s11262-015-1173-1 25663095

[B54] Subangkit S. Mursinah M. Putranto R. H. Seitiawaty V. (2017). Detection of genotype D8 measles virus in Indonesia in 2014. *Health Sci. J. Indonesia* 8 7–11.

[B55] Taira K. Nakamura M. Okano S. Nidaira M. Kudaka J. Itokazu K. (2008). Phylogenetic analysis of nucleoprotein (N) gene of measles viruses prevalent in Okinawa, Japan, during 2003-2007. *Jpn. J. Infect. Dis.* 61 248–250. 18503185

[B56] Takahashi M. Nakayama T. Kashiwagi Y. Takami T. Sonoda S. Yamanaka T. (2000). Single genotype of measles virus is dominant whereas several genotypes of mumps virus are co-circulating. *J. Med. Virol.* 62 278–285. 10.1002/1096-9071(200010)62:2<278::aid-jmv21>3.3.co;2-t 11002259

[B57] Takahashi T. Arima Y. Kinoshita H. Kanou K. Saitoh T. Sunagawa T. (2014). Ongoing increase in measles cases following importations, Japan, March 2014: times of challenge and opportunity. *Western Pac. Surveill. Resp. J.* 5 31–33. 10.5365/wpsar.2014.5.2.001 25077035 PMC4113659

[B58] Takashima Y. Schluter W. W. Mariano K. M. Diorditsa S. De Quiroz Castro M. Ou A. C. (2015). Progress toward measles elimination-Philippines, 1998-2014. *Morb. Mortal Wkly Rep.* 64 357–362. 25856257 PMC4584627

[B59] Tanaka T. Yokoi H. Kobayashi K. Iwanade H. Noguchi Y. Mitsui Y. (2011). First detection of measles virus genotype g3 in a Japanese woman: an imported case. *Jpn. J. Infect. Dis.* 64 262–263. 21617317

[B60] Watanabe A. Kobayashi Y. Shimada T. Yahata Y. Kobayashi A. Kanai M. (2017). Exposure to H1 genotype measles virus at an international airport in Japan on 31 July 2016 results in a measles outbreak. *Western Pac. Surveill. Resp. J.* 8 37–39. 10.5365/wpsar.2016.7.4.007 28409058 PMC5375098

[B61] Watanabe K. Watanabe K. Tazawa T. Kon M. Tamura T. Komase K. (2012). Imported cases of measles in Niigata, Japan in 2011. *Jpn. J. Infect. Dis.* 65 268–270. 10.7883/yoken.65.268 22627313

[B62] World Health Organization [WHO] (2013a). *Global Vaccine Action Plan 2011–2020.* Geneva: WHO.

[B63] World Health Organization [WHO] (2013b). Framework for verifying elimination of measles, and rubella. *Wkly Epidemiol. Rec.* 88 89–99. 23540051

[B64] World Health Organization [WHO] (2015). Genetic diversity of wild-type measles viruses, and the global measles nucleotide surveillance database MeaNS. *Wkly Epidemiol. Rec.* 90 373–380. 26211016

[B65] World Health Organization [WHO] and Public Health England (PHE). (2019). *Measles Nucleotide Surveillance (MeaNS) Database*. Available: http://www.who-measles.org/Public/Web_Front/main.php (accessed December 22 2018).

[B66] World Health Organization [WHO] and Regional Office for the Western Pacific [WPRO] (2013). *Guidelines on Verification Of Measles Elimination in the Western Pacific Region.* Manila: WHO Regional Office for the Western Pacific.

[B67] World Health Organization [WHO] and Regional Office for the Western Pacific [WPRO] (2014). *Third Annual Meeting Of The Regional Verification. Commission for Measles Elimination in the Western Pacific.* Manila: WHO Regional Office for the Western Pacific.

[B68] World Health Organization [WHO] and Regional Office for the Western Pacific [WPRO] (2015). *Fourth Annual Meeting of the Regional Verification. (Commission)for Measles Elimination in the Western Pacific.* Manila: WHO Regional Office for the Western Pacific.

[B69] World Health Organization [WHO] and Regional Office for the Western Pacific [WPRO] (2016). *Fifth Annual Meeting of the Regional Verification. (Commission)for Measles Elimination in the Western Pacific.* Manila: WHO Regional Office for the Western Pacific.

[B70] World Health Organization [WHO], Regional Office for the Western Pacific [WPRO] (2017). *Sixth Annual Meeting of the Regional Verification. (Commission)for Measles Elimination in the Western Pacific.* Beijing: World Health Organization.

[B71] Xu S. Zhang Y. Rivailler P. Wang H. Ji Y. Zhen Z. (2014). Evolutionary genetics of genotype H1 measles viruses in China from 1993 to 2012. *J. Gen. Virol.* 95 1892–1899. 10.1099/vir.0.066746-0 24914068 PMC4135087

[B72] Yagi Y. Higashino H. Yoshida H. Hirokawa H. Okumachi A. Takano M. (2015). The 2014 measles outbreak in Osaka An epidemiological study for the elimination of measles. *Nihon Koshu Eisei Zasshi* 62 566–573. 10.11236/jph.62.9_566 26608046

